# Screen exposure and emotion regulation abilities in 5–6-year-old children: a moderated mediation model

**DOI:** 10.3389/fpsyt.2026.1828704

**Published:** 2026-06-05

**Authors:** Dan Kang, Guanni Jiang, Xiwu Xu

**Affiliations:** 1 School of Education Science, Hunan Normal University, Changsha, Hunan, China; 2 School of Preschool Education, Wuhan City Polytechnic, Wuhan, Hubei, China

**Keywords:** developmental risk, early childhood, emotion regulation, executive function, parent-child interaction, screen exposure

## Abstract

**Introduction:**

Screen exposure is increasingly common in early childhood, yet how it is related to children’s emotion regulation remains insufficiently understood. This study examined the association between screen exposure and emotion regulation in 5–6-year-old children. Executive function was examined as a mediator, and parent-child interaction was included as a moderator.

**Methods:**

A total of 806 children aged 5–6 years and their parents were recruited from four kindergartens in H Province, China, through random cluster sampling. Parents completed questionnaires assessing children’s screen exposure, executive function, emotion regulation, and parent-child interaction. Data were analyzed using Mplus.

**Results:**

Higher screen exposure was associated with lower emotion regulation abilities in children. Executive function partially mediated the association between screen exposure and emotion regulation. Parent-child interaction moderated both the first stage of the “screen exposure—executive function—emotion regulation” pathway and the direct pathway from screen exposure to children’s emotion regulation abilities. Specifically, the negative association between screen exposure and executive function was weaker when parent-child interaction was higher, and the direct association between screen exposure and emotion regulation was no longer significant.

**Discussion:**

The findings suggest that the association between screen exposure and early emotion regulation may be better understood by considering both children’s executive function and the quality of parent-child interaction. These findings may inform family-based guidance on children’s screen use and emotion regulation and provide early evidence relevant to socio-emotional adjustment during adolescence.

## 1 Introduction

Emotion regulation involves the processes that individuals use to manage the generation, experience, and expression of emotions (1). Children begin to show initial signs of emotion regulation around age 2, with this skill’s development being particularly critical between ages 3 and 6. During this period, emotion regulation is a core component of children’s social-emotional development. It is closely related to peer interactions, problem behavior onset, psychological well-being, and academic achievement. Conversely, poor emotion regulation is linked to adverse outcomes, including mental health issues such as anxiety and depression, as well as behavioral difficulties (2). “ China’s Kindergarten Education Guidelines (Trial)” prioritizes “emotional stability and happiness in group life” for preschoolers, while the “China’s National Family Education Guidance Outline (Revised Edition)” emphasizes parental responsibility for children’s emotional development. These policy documents reflect a growing policy concern with the role of family in children’s early development. For children aged 5–6 years, the period before school entry is often accompanied by changes in daily routines, peer interactions, and social expectations. In this period, emotion regulation is closely related to how children adjust to new settings, interact with peers, and form early interpersonal relationships.

Screen exposure has become increasingly prevalent among young children. Pervasive screen use (e.g. TV, video games, and smartphones) has become a major issue for young children’s development. The 2019 China Early Childhood Development Report notes that Chinese preschoolers average 0.72 hours of screen time on school days and 1.61 hours on weekends; a substantial proportion of young children exceed the recommended 2-hour daily limit (3). Zhao et al. (4) further noted that 24% of infants had been exposed to screens before age 1, 76% by age 2, and 78.6% of 3-year-old kindergarten entrants exceeded recommended guidelines. In many families, screens are used to occupy or soothe children, making screen exposure a common part of children’s daily lives. Excessive early childhood screen exposure is thus a prevalent, serious public health concern. The World Health Organization guidelines on physical activity and sedentary behavior indicate that prolonged screen time is associated with elevated risks of childhood obesity, cardiovascular disease, depression, and anxiety (5). Moreover, empirical evidence suggests that excessive screen time in children is associated with poorer sleep quality, motor coordination, and cognitive development (6), with potential adverse effects extending into adolescence and adulthood. Notably, screen time may crowd out opportunities for children to learn emotion regulation strategies, thereby hindering the development of self-regulation (7). Emerging evidence also suggests content-specific differences: non-child-directed content (e.g., adult TV) has been associated with a higher likelihood of emotional problems, whereas educational programming has been associated with a lower likelihood (8). Furthermore, tablet and smartphone use has been linked to increased risks of reduced sleep duration and sleep difficulties (9). Despite this research, the specific mechanisms linking screen exposure to young children’s emotion regulation abilities remain poorly understood. Recent longitudinal meta-analytic evidence further suggests bidirectional associations between screen use and socioemotional problems (10). We therefore hypothesized that higher screen exposure would be associated with lower emotion regulation abilities in young children (H1).

According to emotional cognition theory, emotional development results from the combined effects of external environmental events, individual physiological states, and psychological and cognitive processes. Cognitive processes play an important role in emotional development. The relationship between emotion and cognition is complex, as they jointly influence information processing and the development of executive functions (11). Executive function is important for emotion generation and regulation, and children rely on strategies such as attentional shifting in this process (12). Working memory, a foundational component of executive function, is closely related to cognitive–emotional processes (13) and may be relevant to understanding how screen exposure relates to emotion regulation. The prefrontal cortex supports both executive function and emotion regulation neurobiologically (14). Specifically, the dorsolateral prefrontal cortex is critical for the formation and development of emotion regulation abilities (15) and plays a significant role in inhibitory control (16). Excessive screen exposure in young children has been associated with weaker executive functions (17), which in turn may help explain differences in emotion regulation. Longitudinal evidence links preschool screen exposure to weaker executive functioning (18). During the critical brain development phase, between ages 3 and 6, real-world stimuli are vital for promoting cognitive processing and brain connectivity. Excessive screen exposure may lead to passive processing, inhibition of the cognitive control network, and emotional instability. Research has shown that screen time is associated with poorer executive function and educational outcomes in young children (19), with younger children being more vulnerable (20). Taken together, these findings suggest that executive function may help explain how screen exposure is associated with children’s emotion regulation. We therefore hypothesized that executive function would partially account for the association between screen exposure and emotion regulation in children (H2).

Ecological systems theory suggests that the family environment and atmosphere significantly influence the development of children’s cognitive and emotional abilities, which in turn may affect their psychological health and social development. Positive parent-child interactions not only meet children’s physiological and emotional needs but also help establish secure attachment relationships, thereby fostering healthy emotional development (21). In addition, parental involvement plays a crucial role in children’s development. Parents can support their children’s overall development through joint media engagement by providing cognitive, physical, emotional, and technical support (22). This suggests that screen media, when used as a platform for parent-child activities, may positively influence early learning in cognitive and socio-emotional domains, potentially offsetting the negative associations of screen exposure. Research has shown that children aged 2 to 6 who co-view prosocial television programs with their parents exhibit higher levels of empathy, self-efficacy, and emotional recognition (23). However, how parent-child interaction moderates the relationship between screen exposure, executive function, and emotion regulation requires further investigation. We therefore hypothesized that parent-child interaction would moderate the indirect association between screen exposure and emotion regulation through executive function (H3).

**Figure 1:**
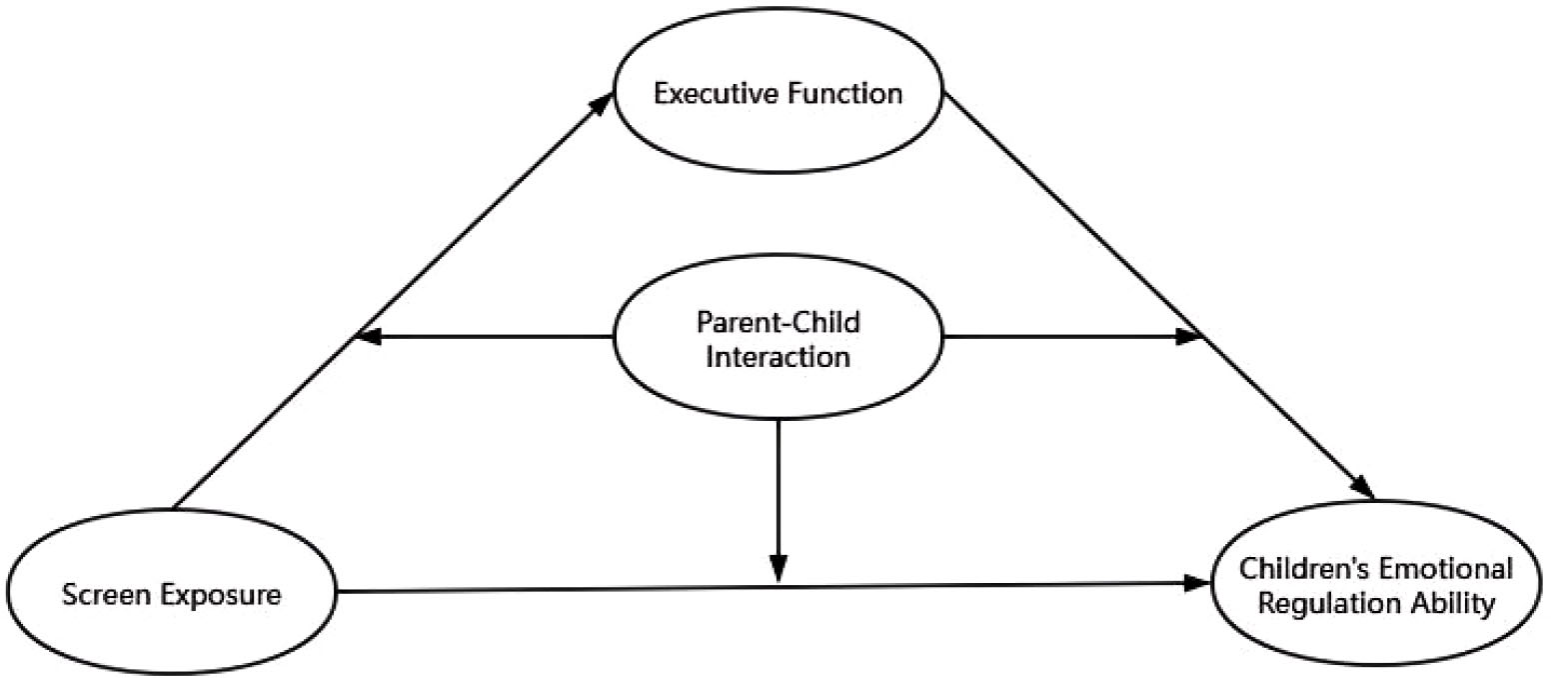
Hypothetical model of executive function as a mediator and parent-child interaction as a moderator.

In summary, the present study examined the associations among screen exposure, executive function, parent–child interaction, and emotion regulation abilities in 5–6-year-old children within a moderated mediation framework. The hypothesized model is shown in **Figure 1**. By testing executive function as a mediating mechanism, this study moves beyond prior work that has mainly focused on the direct association between screen exposure and children’s socio-emotional outcomes. It also examines parent–child interaction as a moderator of both the direct association between screen exposure and emotion regulation and the indirect pathway through executive function. This makes it possible to consider not only whether family context matters, but also where parent–child interaction is most relevant in the model. Methodologically, the study uses latent moderated structural equation modeling to estimate interaction effects at the latent level, which helps reduce the influence of measurement error in moderation analysis. Finally, the study focuses on 5–6-year-old children during the transition from kindergarten to primary school. This is a period when executive function and emotion regulation change rapidly, yet it has received relatively limited attention in screen exposure research.

## 2 Methods

### 2.1 Participants

The required sample size was estimated with G*Power 3.1 using a two-tailed linear multiple regression model, assuming a medium effect size (*f²* = 0.15), *α* = .05, and power (1 − *β*) = .95. The analysis suggested that at least 383 participants were needed. Participants were recruited through random cluster sampling from four kindergartens in H Province, China. In total, 884 questionnaires were returned, and 806 were retained as valid, corresponding to a valid response rate of 91.0%. The final sample consisted of 420 boys (52.1%) and 386 girls (47.9%), the average age was 5.75 years (SD = 0.43). Regarding the family structure, 24.9% of the children were only children, 71.9% were from two-child families, and 3.2% had three or more siblings. With respect to parental education, 76.5% of fathers and 70.4% of mothers had graduated from college and higher. On the other hand, only 1.6% of fathers and 2.0% of mothers had completed elementary or junior secondary education, while 7.2% of fathers and 5.8% of mothers had completed senior secondary education. Data were collected by class. Before the survey was administered, parents were informed of the purpose of the study and assured that their responses would be kept confidential. Ethical approval for the study was obtained from the Ethics Review Committee of the relevant institutions.

### 2.2 Measurement instruments

#### 2.2.1 Multimedia usage questionnaire for children aged 3–6 years

This study used the “Multimedia Usage Questionnaire for Children Aged 3–6 Years,” developed by Li Minyi’s team in 2014 (24). This questionnaire was adapted from numerous international studies on children’s multimedia usage and translated and revised to suit the Chinese context. The questionnaire had 10 items and 93 subitems divided into four sections, with data collected through parent reports. To better understand children’s screen exposure, 17 items related to “daily screen exposure time” and “frequency of multimedia activities” were selected. The Cronbach’s α for this study was 0.95, and the reported validity index was 0.86. Confirmatory factor analysis indicated a good model fit (*χ²/df* = 2.612, CFI = 0.989, TLI = 0.987, RMSEA = 0.045), making it suitable for assessing children’s screen exposure.

#### 2.2.2 Children’s executive function scale

Executive function was measured using the Childhood Executive Functioning Inventory, which is based on Barkley’s hybrid model and was originally developed by Thorell and Nyberg (25). The Chinese version was translated and adapted by Wei Wei et al. (26). The scale includes 24 items rated on a 5-point scale and covers three dimensions: working memory, regulation ability, and inhibitory control. In the original scoring system, higher scores indicate weaker abilities and more severe difficulties. In the Chinese version, the internal consistency coefficients for the three dimensions were 0.89, 0.78, and 0.71, respectively. For ease of interpretation in the present study, reverse scoring was applied so that higher scores reflected better executive function. In the current sample, Cronbach’s α was 0.94, the reported validity index was 0.91, and confirmatory factor analysis showed a good model fit (*χ²/df* = 1.988, CFI = 0.976, TLI = 0.973, RMSEA = 0.035), supporting the suitability of the scale for assessing children’s executive function.

#### 2.2.3 Emotion regulation checklist

The Emotion Regulation Checklist (ERC) developed by Shields and Cicchetti (27), is widely used to assess children’s emotion regulation abilities. The scale consists of 24 items, scored from 1 to 4 across two dimensions, and was completed by parents in the present study. This study used the revised version of Zhu Jingjing et al. (28). The revised ERC had internal consistency coefficients of 0.84 and 0.91 for the respective dimensions, with a total internal consistency of 0.81. The revised checklist includes eight and 13 items for emotion regulation and emotional instability dimensions, respectively, with two and four reverse-scored items. For data processing, the emotional instability dimension was reverse-scored, with higher scores indicating stronger emotion regulation abilities. In this study, Cronbach’s α was 0.97, the reported validity index was 0.84, and confirmatory factor analysis indicated a good model fit (*χ²/df* = 3.163, CFI = 0.976, TLI = 0.974, RMSEA = 0.052), making it suitable for assessing children’s emotion regulation abilities.

#### 2.2.4 Parent-child interaction scale

The Parent–Child Interaction Scale (BPCIS) was used to assess the quality of parent–child interactions. The Chinese version was translated and adapted for Chinese families by Lai Jingjing (29) and Zhang Yao (30). The scale includes 17 items across five dimensions, with 13 positive and 4 negative interaction items rated on a 5-point scale, where higher scores indicate better parent-child interaction quality. The scale demonstrated good reliability and validity in the Chinese cultural context, with an overall Cronbach’s alpha of 0.97. In this study, Cronbach’s α was 0.94, the reported validity index was 0.83, and confirmatory factor analysis indicated a good model fit: *χ²/df* = 1.587, CFI = 0.993, TLI = 0.991, RMSEA = 0.027, making it suitable for assessing the quality of parent-child interactions.

### 2.3 Procedure and data processing

Data were analyzed using SPSS 26.0 and Mplus 8.3. The moderated mediation model was tested in Mplus using the latent moderated structural equations (LMS) approach (31). Compared with manifest-variable methods such as PROCESS, LMS estimates interactions at the latent level and accounts for measurement error, thereby reducing the risk of underestimating moderation effects (32). Because LMS does not provide conventional fit indices for models with latent interaction terms, a model without interaction terms, Model 0 (M0), was first estimated and evaluated for model fit. Latent interaction terms were then added to form Model 1 (M1), and a log-likelihood ratio test was used to determine whether the addition of the latent interaction terms significantly improved model fit (33).Fit indices such as CFI, TLI, and RMSEA were therefore reported for M0 but not for M1.

The study included several scales to measure variables, including the Screen Exposure Scale, Executive Function Scale, Emotion Regulation Checklist, and Parent-Child Interaction Quality Scale, all of which are rated by parents. To minimize the risk of common method bias, both procedural and statistical steps were taken. During questionnaire administration, participants were informed that their responses would remain anonymous and confidential. As an initial check, Harman’s single-factor test was conducted. The results showed that 11 principal components were extracted without rotation, with the first principal component explaining 28.09% of the total variance, which was below the 40% threshold (34). Moreover, we assessed potential common method variance through confirmatory factor analysis, comparing a baseline model without a method factor with a model that included an unmeasured latent method construct (ULMC). The baseline model showed a good fit to the data (*χ²* = 4770.116, *df* = 2984, CFI = 0.966, TLI = 0.965, RMSEA = 0.027, 90% *CI* [0.026, 0.029], SRMR = 0.043). After adding the latent method factor, the model fit was *χ²* = 4649.405, df = 2979, CFI = 0.968, TLI = 0.967, RMSEA = 0.026, 90% *CI* [0.025, 0.028], and SRMR = 0.040. Compared with the baseline model, the improvement in model fit was small (ΔCFI = 0.002 < 0.01, ΔTLI = 0.002 < 0.01, ΔRMSEA = -0.001 < 0.015, ΔSRMR = -0.003 < 0.03), suggesting that common method variance was unlikely to pose a substantial threat to the findings. Nevertheless, since all core variables were assessed by parents, common method bias cannot be completely ruled out.

## 3 Results

### 3.1 Descriptive statistics and correlation analysis

Correlation analysis was performed among children’s screen exposure, executive function, emotion regulation abilities, and parent-child interaction. The results showed that screen exposure was significantly and negatively correlated with children’s executive function, emotion regulation abilities, and parent-child interaction. Conversely, executive function, emotion regulation abilities, and parent-child interaction were positively correlated with each other. The detailed statistics are presented in **Table 1**.

**Table 1:** Means, standard deviations, and correlation matrix of variables (N = 806).

Variables	*M*	*SD*	Gender	Screen exposure	Executive function	Emotion regulation ability	Parent-child interaction
1.Gender	—	—	—				
2.Screen Exposure	3.03	1.30	−.021	—			
3.Executive Function	3.04	0.72	.062	*−*.381^**^	—		
4.Emotion Regulation Ability	2.79	0.78	.050	*−*.332^**^	.504^**^	—	
5.Parent-Child Interaction	3.28	0.92	.003	−.154^**^	.258^**^	.253^**^	—

*p <.05; **p <.01; ***p <.001.

### 3.2 The relationship between screen exposure and emotion regulation ability: moderated mediation effects analysis

A moderated mediation model was tested in Mplus 8.3 to examine the associations among screen exposure, executive function, parent-child interaction, and children’s emotion regulation abilities, with executive function specified as the mediator and parent-child interaction as the moderator of both the direct and indirect pathways (35). After controlling for gender, the analysis showed that the measurement model had a good fit: *χ²/df* = 1.672, CFI = 0.990, TLI = 0.987, RMSEA = 0.029, and SRMR = 0.025. As shown in **Table 2**, the basic model (M0) fit well. Screen exposure was negatively associated with children’s emotion regulation abilities (*β* = -0.168, *p* < 0.05), consistent with Hypothesis H1. Additionally, executive function was positively related to children’s emotion regulation abilities (*β* = 0.450, *p* < 0.001), whereas screen exposure was negatively associated with executive function (*β* = -0.546, *p* < 0.001). The indirect association through executive function was significant (*β* = -0.25, *p* < 0.001), accounting for 67.9% of the total effect, thus providing support for Hypothesis H2.

**Table 2:** Results of the latent moderated structural equation models (M0 and M1).

Model	Path	*β*	*SE*	*p*	95%CI
M0	Screen exposure → Emotion regulation ability	−.168^*^	.072	.021	[−.314, −.020]
	Executive Function → Emotion regulation ability	.450^***^	.056	<.001	[.336, .557]
	Screen exposure →Executive Function	−.546^***^	.050	<.001	[−.647, −450]
M0	Screen exposure → Emotion regulation ability	−.168^*^	.072	.021	[−.314, −.020]
	Screen exposure → Emotion regulation ability	−.129^***^	.045	<.001	[−.218, −.041]
	Executive Function → Emotion regulation ability	.261^***^	.048	<.001	[.167,.356]
	Screen exposure → Executive Function	−.262^***^	.045	<.001	[−.350, −.174]
	Parent-Child Interaction → Executive Function	.206^***^	.048	<.001	[.111,.301]
	Parent-Child Interaction → Emotion regulation Ability	.015	.038	.694	[−.060,.090]
	Screen Exposure × Parent-Child Interaction → Executive Function	.145^**^	.046	.003	[.048,.243]
	Screen Exposure × Parent-Child Interaction →Emotion regulation Ability	.103^*^	.050	.039	[.005,.201]
	Executive Function × Parent-Child Interaction → Emotion regulation Ability	.088	.049	.072	[−.008,.183]

M0 model fit: *χ²/df* = 1.672, CFI = 0.990, TLI = 0.987, RMSEA = 0.029, SRMR = 0.025. Log-likelihood ratio test comparing M1 to M0: *D*(df = 3) = 98.75, *p* <.001. *p <.05; **p <.01; ***p <.001.

**Figure 2:**
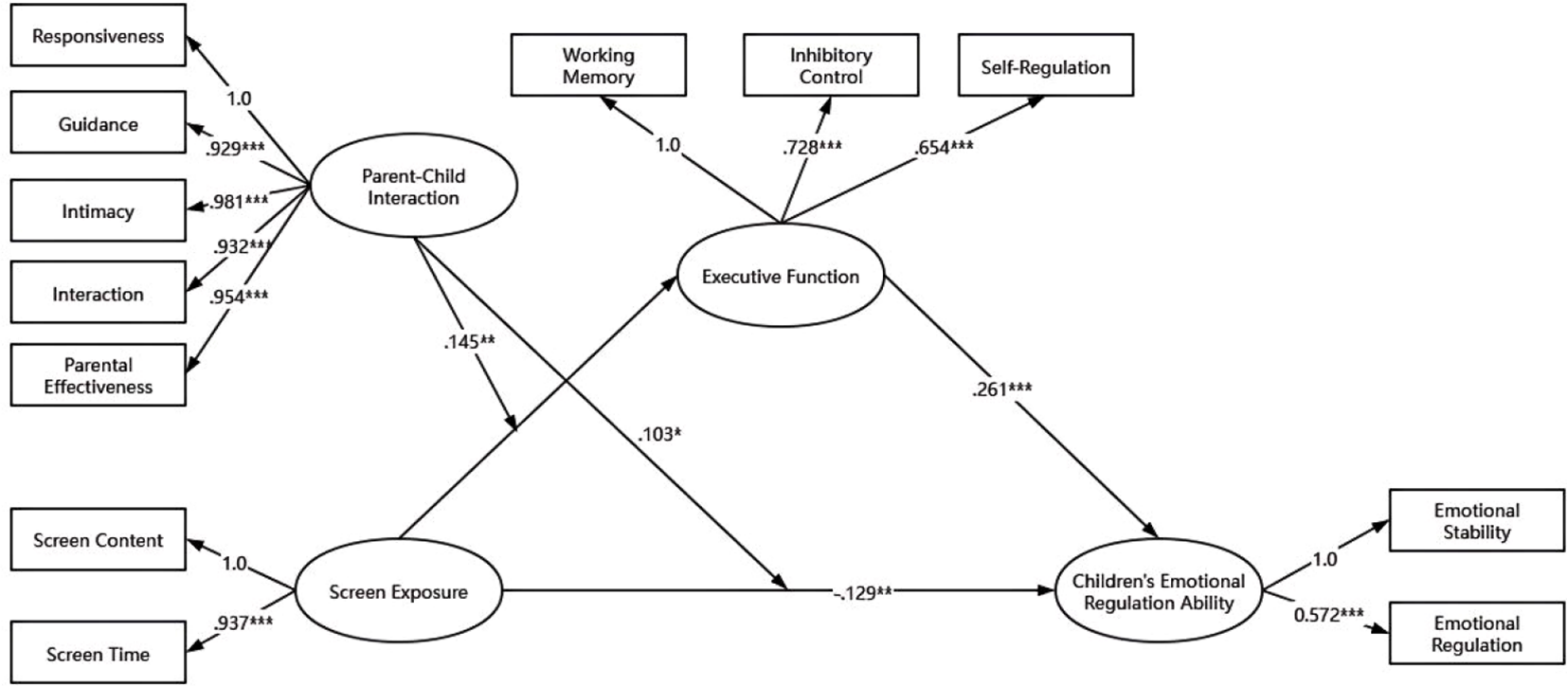
Structural equation model testing the moderated mediation model.

Building on the basic model M0, moderated mediation model M1 was developed. M1 included latent interaction terms between screen exposure and parent-child interaction, as well as between executive function and parent-child interaction. A log-likelihood ratio test revealed that D(*df* = 3) = 98.75, *p* < 0.001, indicating that M1 fit the data better than M0, supporting the moderated mediation model. Specifically, M1 analysis showed that the interaction between screen exposure and parent-child interaction was significantly associated with children’s emotion regulation abilities (*β* = 0.103, *p* < 0.05) and executive function (*β* = 0.145, *p* < 0.01). However, the interaction between executive function and parent-child interaction was not significantly associated with children’s emotion regulation abilities (*β* =0.088, *p* > 0.05). These results partially supported Hypothesis H3, suggesting that parent-child interaction mainly moderates the first half of the mediation path and the direct association. The structural equation model is shown in **Figure 2**.

**Figure 3:**
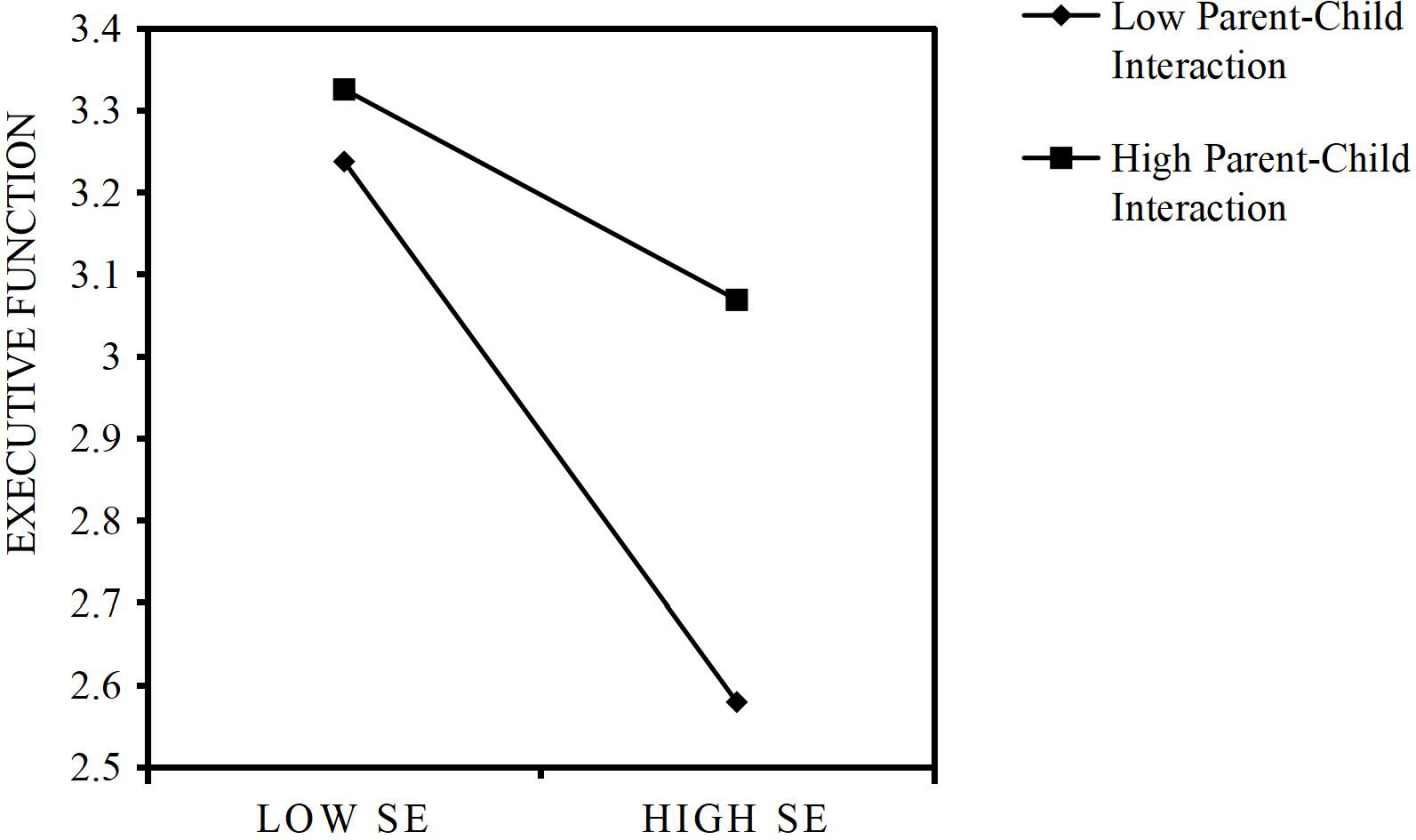
The moderating role of parent-child interaction in the relationship between screen exposure and executive function.

**Figure 4:**
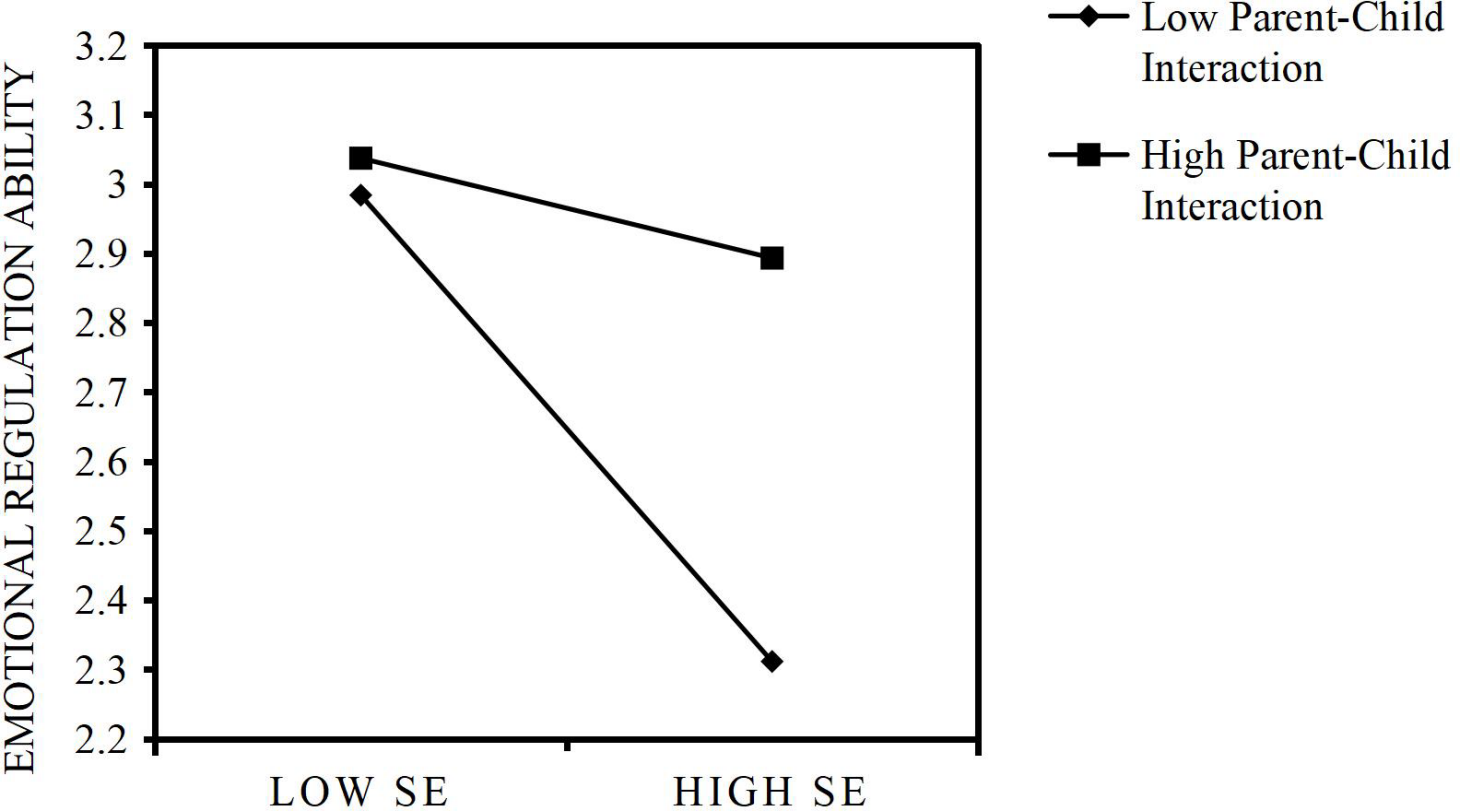
The moderating role of parent-child interaction in the relationship between screen exposure and emotion regulation ability. SE, screen exposure.

To further explore how parent-child interaction influences the association between screen exposure and children’s emotion regulation abilities, a simple slope analysis was conducted by categorizing parent-child interaction into high and low groups based on ± 1 SD. The results, depicted in **Figure 3** and **4**, showed that when the quality of parent-child interaction is low, screen exposure was significantly and negatively associated with children’s executive function (*β* = -0.253, *t* = -11.30, *p* < 0.001) and emotion regulation abilities (*β* = -0.141, *t* =- 3.44, *p* < 0.001). However, when the quality of parent-child interaction is high, the negative association between screen exposure and executive function remains significant but is weakened (*β* = -0.099, *t* = -3.68, *p* < 0.01), and its association with emotion regulation abilities is not significant (*β* = -0.018, *t* = -0.597, *p* > 0.05). These results indicate that the negative association between screen exposure and children’s executive function was weaker at higher levels of parent-child interaction, whereas the association between screen exposure and emotion regulation abilities was no longer significant.

## 4 Discussion

The results of this study showed that higher screen exposure was associated with lower emotion regulation abilities in children. Executive function partly mediated this association, suggesting that cognitive self-regulation may be one pathway linking screen exposure with emotion regulation. In addition, parent-child interaction moderated both the direct association between screen exposure and emotion regulation and the first stage of the mediating pathway through executive function. These findings suggest that parent-child interaction may serve a buffering role in children’s development by shaping both executive function and emotion regulation. The implications for family- and kindergarten-based strategies are discussed below.

### 4.1 The relationship between screen exposure and children’s emotion regulation abilities

The present findings suggest that higher screen exposure was associated with lower emotion regulation abilities in children, which is consistent with Hypothesis H1. The Differential Susceptibility to Media Effects Model (DSMM) suggests that the inappropriate use of screen media may be associated with adverse outcomes in children. Inappropriate usage includes excessive screen time and unsuitable content, children’s media experiences may be related to developmental outcomes through cognitive, emotional, and social processes. One possible explanation is that excessive screen time may be associated with greater emotional instability among children. According to the Social Withdrawal Hypothesis, excessive screen time may limit children’s bidirectional interactions with their peers and parents (36). Greater immersion in screen media may in turn limit social engagement and be associated with withdrawal-related negative or anxious emotions. Previous research has suggested that screen time exceeding 1–2 hours per day may be associated with more negative emotional outcomes (6).

Moreover, insufficient sleep due to screen exposure has been linked to irritability and emotional instability. For instance, tablet and smartphone use has been linked to increased risks of reduced sleep duration and sleep difficulties (9), both of which may be relevant to children’s emotional functioning. Neurobiological research has suggested that prolonged screen time may be associated with altered brain functioning in frontal regions involved in self-regulation (37). Complementing this, neuroimaging research has shown that parent-child reading (≥15 minutes/day) may attenuate screen exposure’s adverse impact on brain white matter integrity, highlighting protective factors’ potential role in neural development (38).In addition, the abstract and fast-paced nature of screen content may limit young children’s opportunities to authentically experience and process complex emotions, potentially hindering the development of emotion regulation skills.

### 4.2 The mediating role of executive function

These results confirmed H2 in terms of the partial mediation role played by executive function in the connection between screen exposure and children’s emotion regulation skills. These findings align with the principles of the DSMM, which postulates that media experiences are linked with developmental outcomes among children via the mechanisms of cognition and emotions within an individual. In the present research, executive function seems to be one of those cognitive mechanisms that link screen exposure and children’s emotion regulation.

One possible explanation for this could be that emotion regulation is based on the functioning of some cognitive skills which are provided by executive functions. Working memory, selective attention, and inhibition control can facilitate emotional awareness, situational rule awareness, inhibition of emotional reactions, and adaptive behaviors when facing emotionally challenging situations. Research shows that screen exposure has been linked to both internal and external behavioral problems in children (39). There is evidence from studies that examined young children where it was found that higher executive functioning has an association with better emotion regulation ability (40).

Attention and executive function may also play an important role in this context. Executive function refers to the inhibition of distracting stimuli, maintaining attention towards goal-directed behavior, and flexibility in task-switching or set-shifting (41). Prolonged screen use could mean fewer chances for children to engage in sustained and voluntary attention in their daily activities. Excessive screen time has also been connected with poor physical well-being and psychological states, as well as sleep disturbances (42), which may affect self-regulation among children. Neurodevelopmental research has shown that early screen use can affect EEG measurements and cognitive performance (43).

For this reason, the relationship between screen exposure and the emotion regulation of children cannot be explained solely through behavior. It may also be necessary to look into cognitive self-regulation. The basis for this explanation comes from research revealing the strong link between executive functions and emotion regulation. Executive function has been discussed as a mechanism related to improvements in emotion regulation (44), and recent longitudinal evidence among preschool children suggests a reciprocal relation between executive function and emotion regulation (45). Overall, weaker executive function may represent one possible pathway linking higher screen exposure with poorer emotion regulation. This finding highlights the need to pay closer attention to children’s cognitive self-regulation in research on screen exposure and socio-emotional development.

### 4.3 The moderating role of parent-child interaction

The results partially support Hypothesis H3, indicating that parent-child interaction moderates the first stage of the indirect pathway (“screen exposure-executive function-emotion regulation abilities”) and the direct pathway between screen exposure and children’s emotion regulation abilities. This suggests high-quality parent-child interaction may function as a protective factor for executive function and emotion regulation in the context of screen exposure. Positive parent–child interaction has been associated with early cognitive and emotional development (46), which may support the development of emotion regulation.

High-quality parent-child interactions strengthen parent-child attachment and emotional security. Attachment theory highlights the importance of early secure attachment in social-emotional development (47). Notably, the quality of parent-child interaction—including parental emotional responsiveness—has been associated with children’s behavioral and emotional outcomes (48). Consistent with the present findings, supportive emotional expression may help buffer the association between screen exposure and poorer emotion regulation by complementing children’s executive function. It may also help children distinguish screen content from real experiences: parents can guide children in interpreting content, prioritize educational materials, and provide emotional support, thereby fostering the development of more adaptive emotion regulation strategies. Lastly, parent-child interaction enhances language, working memory, and executive function through imitation, problem-solving, and play. According to executive function theory (41), executive function includes higher-order cognitive processes critical for learning, attention control, and goal-directed behavior. High-quality parent-child interactions provide contexts to practice executive function, helping children better handle cognitive and emotional challenges related to screen exposure.

In conclusion, parent-child interaction—through parental emotional expression and guidance toward positive screen content—may provide emotional support and a cognitive-behavioral framework to buffer screen exposure’s negative associations. Parental technoference—device use that disrupts interaction—has been associated with poorer socioemotional outcomes and increased problematic media use in children (49). An important finding was that parent–child interaction moderated the first part of the indirect pathway as well as the direct association, but not the second part of the pathway (executive function → emotion regulation). This uneven pattern may indicate that parent–child interaction plays a stronger role in shaping the conditions under which screen exposure is linked to children’s cognitive and emotional resources than in strengthening the extent to which executive function is associated with emotion regulation. From a practical perspective, this may mean that more active forms of parent–child engagement—such as shared reading, discussion of screen content, and collaborative play—are likely to be more helpful than passive co-viewing in reducing the negative associations of screen exposure (22). At the same time, the fact that this protective role was only partial suggests that parent–child interaction alone may not be sufficient, and that broader ecological support may also be needed.

### 4.4 Implications, limitations, and future directions

This study examined how screen exposure, executive function, parent-child interaction, and children’s emotion regulation abilities are related to one another. By including both executive function and parent-child interaction, the study provides a more detailed view of the link between screen exposure and emotion regulation in early childhood. The findings suggest that healthy media use cannot be supported simply by reducing screen time. Content quality, children’s self-regulatory capacities, and everyday family interaction also deserve attention. For families and kindergartens, guidance on screen use should therefore be combined with efforts to strengthen children’s self-regulation and promote more responsive parent-child interaction.

Several limitations should be noted. First, the sample included urban children only, so the findings may not fully apply to children in rural areas. Second, as a cross-sectional study, it did not capture the longitudinal effects of screen exposure on emotion regulation abilities over time. Third, all core variables were reported by parents, although procedural and statistical controls were used, single-informant bias and common method bias cannot be completely ruled out. Moreover, the findings should also be interpreted in light of mixed evidence on the association between screen exposure and executive function. For example, some meta-analytic evidence has reported a null aggregate effect among children under age 6 (50), which may be partly due to differences in how screen exposure is measured. Finally, this study focused mainly on the quantity of screen exposure. It did not examine content type, context of use, or patterns of parent-child co-use in depth, although these factors may also be relevant to children’s emotion regulation. Future studies could use longitudinal or cross-lagged designs to clarify the direction of these associations. They could also include multi-informant or observational measures and examine how different screen content and family media practices are related to children’s emotion regulation over time.

## 5 Conclusion

1. Screen exposure was negatively associated with children’s emotion-regulation abilities.
2. Executive function partially mediated the association between screen exposure and children’s emotion regulation abilities.
3. Parent-child interaction moderated both the first stage of the indirect pathway linking screen exposure, executive function, and emotion regulation and the direct association between screen exposure and children’s emotion regulation abilities.

## Data Availability

The raw data supporting the conclusions of this article will be made available by the authors, without undue reservation.
